# Patient and provider perceptions of a community‐based accompaniment intervention for adolescents transitioning to adult HIV care in urban Peru: a qualitative analysis

**DOI:** 10.1002/jia2.26019

**Published:** 2022-10-17

**Authors:** Jerome T. Galea, Milagros Wong, Brennan Ninesling, Alicia Ramos, Liz Senador, Hugo Sanchez, Lenka Kolevic, Eduardo Matos, Eduardo Sanchez, Renato A. Errea, Andrew Lindeborg, Carlos Benites, Leonid Lecca, Sonya Shin, Molly F. Franke

**Affiliations:** ^1^ School of Social Work College of Behavioral and Community Sciences University of South Florida Tampa Florida USA; ^2^ College of Public Health University of South Florida Tampa Florida USA; ^3^ Department of Global Health and Social Medicine Harvard Medical School Boston Massachusetts USA; ^4^ Socios En Salud Sucursal Peru Lima Peru; ^5^ Morsani College of Medicine University of South Florida Tampa Florida USA; ^6^ Epicentro Lima Peru; ^7^ Servicio de Infectologia Instituto Nacional del Salud del Niño Lima Peru; ^8^ Programa de ITS VIH/SIDA y hepatitis Ministerio de Salud Lima Peru; ^9^ Servicio de Infectología Hospital Nacional Arzobispo Loayza Lima Peru; ^10^ Servicio de Enfermedades Infecciosas y Tropicales Hospital Nacional Hipólito Unanue Lima Peru; ^11^ Division of Global Health Equity Brigham and Women's Hospital Boston Massachusetts USA

**Keywords:** HIV, adolescents, adherence, transition, Peru, community‐based accompaniment

## Abstract

**Introduction:**

Adolescents living with HIV (ALWH) experience higher mortality rates compared to other age groups, exacerbated by the suboptimal transition from paediatric to adult HIV care, during which decreased adherence to antiretroviral therapy (ART) and unsuppressed viremia are frequent. Care transition—a process lasting months or years—ideally prepares ALWH for adult care and can be improved by interventions that are youth‐friendly and address psychosocial issues affecting ART adherence; however, such interventions are infrequently operationalized. Community‐based accompaniment (CBA), in which laypeople provide individualized support and health system navigation, can improve health outcomes among adults with HIV. Here, we describe patient and provider perceptions of a novel HIV CBA intervention called “PASEO” for ALWH in Lima, Peru.

**Methods:**

PASEO consisted of six core elements designed to support ALWH during and after the transition to adult HIV care. During 2019–2021, community‐based health workers provided tailored accompaniment for ALWH aged 15–21 years over 9 months, after which adolescent participants were invited to provide feedback in a focus group or in‐depth interview. HIV care personnel were also interviewed to understand their perspectives on PASEO. A semi‐structured interview guide probing known acceptability constructs was used. Qualitative data were analysed using a framework analysis approach and emergent themes were summarized with illustrative quotes.

**Results:**

We conducted five focus groups and 11 in‐depth interviews among *N* = 26 ALWH and nine key‐informant interviews with HIV care personnel. ALWH participants included males, females and one transgender female, and those with both early childhood and recent HIV infection. ALWH praised PASEO, attributing increased ART adherence to the project. Improved mental health, independence, self‐acceptance and knowledge on how to manage their HIV were frequently cited. HIV professionals similarly voiced strong support for PASEO. Both ALWH and HIV professionals expressed hope that PASEO would be scaled. HIV professionals voiced concerns regarding financing PASEO in the future.

**Conclusions:**

A multicomponent CBA intervention to increase ART adherence among ALWH in Peru was highly acceptable by ALWH and HIV programme personnel. Future research should determine the efficacy and economic impact of the intervention.

## 1 INTRODUCTION

HIV is a manageable disease and mortality should be rare; however, adolescents aged 10–19 living with HIV (ALWH)—about 1.8 million globally [1]—experience worse health outcomes than other age groups. HIV is the second leading cause of death among adolescents aged 10–19 years [1, 2, 3], and among youth aged 15–19 years, HIV‐associated mortality is rising [4]. For ALWH, becoming an adult is an especially precarious period: in addition to biopsychosocial changes, ALWH also transition from paediatric to adult HIV care and are frequently “lost in transition,” especially in low‐resource settings [5].

Care transition is a process lasting months or years, and, if unsuccessful, poses risks to HIV care engagement, retention [6] and mental health [7]. Ideally, ALWH are virally suppressed before, during and after transition [8]. Viral suppression pre‐transition is vital; a 2020 systematic review (*N* = 24 studies) of ALWH's care transition experiences found the worst health outcomes among those with unsuppressed viremia [9]. However, in ALWH, viral suppression rates are disappointing. For example, among a cohort of 1411 U.S. ALWH aged 13–29 years, after a median follow‐up of 5 months, 34% were retained in HIV care and started antiretroviral therapy (ART), and 12% achieved viral suppression [10]. In the 2017 South African National HIV Prevalence, Incidence, Behaviour and Communication Survey, among *N* = 398 ALWH aged 10–19 years with viral load results, 48.9% were virally suppressed [11].

Research on the factors affecting care transition among ALHW is rapidly expanding [11, 12, 13, 14, 15, 16, 17], though data are primarily from Africa, the United States and Europe. Interventions that are individualized, youth‐friendly, collaborative between youth and medical providers, and responsive to mental/psychosocial needs are consistently noted [18, 19, 20, 21, 22, 23]. Several studies highlight ALWH's transition experiences, emphasizing core competencies to manage HIV autonomously [24, 25]. Only one review included Latin America [26], finding that structural‐ and individual‐level barriers (e.g. lack of family support, stigma, poverty and later HIV disclosure) negatively affect ALWH's treatment outcomes.

Despite the evidence on facilitators of successful care transition, research improving ALWH's experiences is scant. An approach that enhances HIV outcomes among adults—community‐based accompaniment (CBA)—holds promise [27]. CBA trains lay people to provide support, education and assistance navigating the HIV care system. For example, adults living with HIV in Peru and Rwanda receiving HIV CBA had higher suppressed viremia rates and improved mental and social health outcomes relative to control groups [28, 29, 30, 31]; however, CBA for ALWH transitioning to adult HIV care is untested.

In Peru, approximately 98,000 people live with HIV [32], of which 26,642 are 15–24 years of age [33]. ALWH in Peru comprise youth with perinatally and recently acquired HIV, young men who have sex with men and transgender women; the epidemic is concentrated among the latter two groups, with those aged 20–24 years experiencing the highest HIV incidence rates compared to other age groups [33]. Here, we describe ALWH's and HIV providers’ perspectives of a CBA intervention for ALWH transitioning to adult HIV care in Lima, Peru.

## 2 METHODS

### 2.1 Participants and procedures

During 2019–2021, we pilot‐tested a CBA intervention called “PASEO” implemented by the community‐based organization Socios En Salud (SES) in Lima. Building on previous ART adherence research among Peruvian ALWH [34], we recruited ALWH aged 15–21 years; commencing or taking ART; preparing to transition to adult HIV care; and residing in Lima. ALWH disengaged from adult HIV care were also eligible. Recruitment occurred from October 2019 to January 2020 at three public hospitals providing HIV care where clinicians consecutively referred eligible ALWH to study staff.

**Figure 1:**
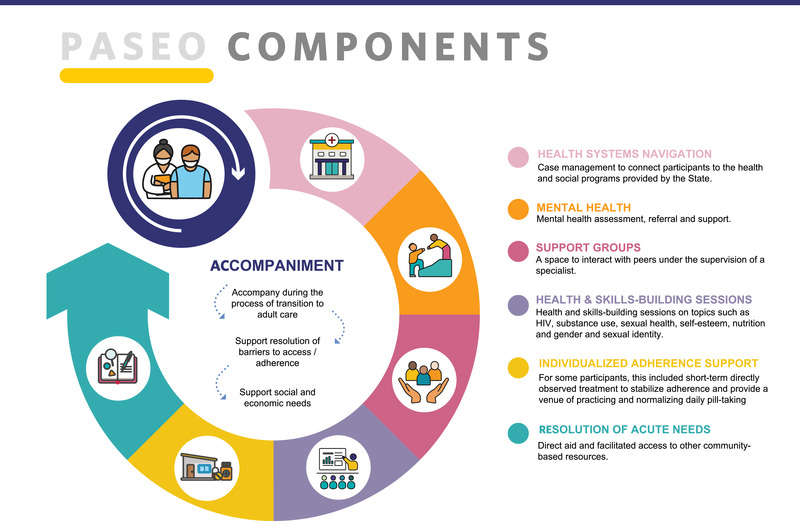
PASEO core components. The PASEO intervention provided community‐based accompaniment to adolescents living with HIV and focused efforts on six core components.

The PASEO intervention and feasibility results are detailed elsewhere [35]. Briefly, PASEO consisted of six core components (Figure 1) delivered over 9 months (a 6‐month intensive phase followed by a 3‐month step‐down phase) by paid entry‐level or lay‐health workers: 1. Health system navigation and clinic visit accompaniment (at least monthly); 2. Social support groups (monthly); 3. Screening and referral to mental health services; 4. Resolution of acute needs (e.g. psychosocial, medical, housing and transportation); 5. Health education and skills‐building (monthly); and 6. Individualized adherence support, including directly observed therapy (DOT) (as needed). PASEO addressed individual circumstances, with the goal of building ALWH's knowledge, skills and confidence to self‐manage HIV care. For example, if an ALWH was experiencing unstable housing, housing support was coordinated (core component 4). Likewise, for ALWH experiencing mental distress or substance use issues, linkage to care was provided (core component 6).

Thirty ALWH naïve to SES participated, comprising those with early childhood or recently acquired HIV, cisgender males and females, males identifying as homosexual and a transgender female. When the SARS‐CoV‐2 (COVID‐19) pandemic required mandatory stay‐at‐home orders (March 2020), participants had received between 1.4 and 5.3 months of the intervention, after which we implemented virtual delivery of the intervention (detailed in [35, 36]). Essentially, all previously in‐person study activities were conducted via telephone/videoconferencing (participants were provided data credits). For example, ALWH received weekly accompaniment telephone calls to coordinate ART collection (e.g. support navigating COVID‐19 transportation restrictions). For ALWH receiving DOT, video calls were implemented. Secure virtual spaces were harnessed to continue the support groups (core component 2) and promote participants’ interaction, socialization and education which were stressed during the acute phase of COVID‐19 [34].

An ALWH‐comprised Youth Advisory Board guided PASEO, and ethics approvals in Peru and the United States covering the study intervention, including qualitative data collection, were obtained at enrolment. Written informed consent was obtained from adolescents aged ≥18 years and from guardian(s) of adolescents aged <18 years with informed assent. Consent was waived for adolescents aged <18 years without guardians; they provided informed assent.

### 2.2 Data collection

Thirty ALWH participated in PASEO, and all were invited to participate in a focus group or in‐depth interview at intervention/study completion. MW, PASEO study coordinator and nurse trained in qualitative methods, collected the data in Spanish. Focus group and individual in‐depth interview composition was determined *a priori*, arriving at five different participant experiences/types: A. ALWH who regularly participated in all PASEO intervention components; B. ALWH who did not regularly participate in all PASEO intervention components; C. Pregnant ALWH; D. ALWH who transitioned from paediatric to adult care during PASEO; and E. ALWH initiating ART just before PASEO. In‐depth interviewees were selected by convenience to gain a deeper understanding of participant experiences. Additionally, we conducted key informant interviews with HIV care personnel (physicians, peer counsellors, psychologists and programme officials) who referred adolescents to PASEO and providers from whom PASEO participants received services.

The focus groups, in‐depth and key‐informant interviews followed semi‐structured interview guides informed by eight key intervention acceptability focus areas: acceptability, demand, implementation, practicality, adaptation, integration, expansion and limited efficacy [37] (Table 1). Prior to the focus groups and interviews, participants were asked to find a private, distraction‐free space, and reminded to prevent others (e.g. household members) from hearing or seeing interviews; headphones were provided to participants for increased privacy. The study staff was likewise situated in a private space. Data were collected via secure/HIPAA‐compliant videoconferencing; for three participants, interviews occurred in real‐time, via encrypted text messages because they relocated outside Lima and lacked internet. Video was used whenever possible; however, cameras were switched off during poor audio quality. Procedures for maintaining research continuity during COVID‐19 were followed, including environmental issues (discussing/ensuring distraction‐free privacy), using professional attire; and readiness to address technical issues [38]. Interviews and focus groups lasted approximately 60‐minutes, were recorded, transcribed verbatim (for interviews conducted via chat, the chat transcript was the source document) and analysed using Dedoose [39].

**Table 1:** Areas of focus, outcomes of interest and illustrative questions for qualitative data collection guides used in focus groups and interviews to assess the acceptability of the PASEO intervention**^a^**

Area of focus and outcomes of interest^a^		Illustrative questions from qualitative guides for ALWH (focus groups and interviews) and healthcare personnel (interviews)
Acceptability	Areas of focus: To what extent is a new idea, programme, process or measure judged as suitable, satisfying or attractive to programme deliverers? To programme recipients? Outcomes of interest: Satisfaction, intent to continue to use, perceived appropriateness	For adolescents: The first three words to describe what I think about PASEO are: What did you like most about PASEO? What did you like the least about PASEO? What is your opinion and/or experience with the: …support provided to enrol in health insurance? …reminders and accompaniment to hospital visits? …home visits by the health promoter? …social support groups? …support in managing the side effects of medications? …the duration of the intervention? For healthcare personnel: What are your general impressions of the intervention? What did you like most about the intervention? What did you like the least about the intervention? What is your opinion about the duration of the intervention?
Demand	Areas of focus: To what extent is a new idea, programme, process or measure likely to be used (i.e. how much demand is likely to exist?) Outcomes of interest: Fit within organizational culture, perceived positive or negative effects on the organization, actual use, expressed interest or intention to use, perceived demand	For healthcare personnel: How important was the intervention for adolescents living with HIV who are transitioning to adult care? Did any of the adolescents talk to you? What did they say? For whom do you think the intervention is not useful? What additional information would you have liked to see covered in the intervention?
Implementation	Areas of focus: To what extent can a new idea, programme, process or measure be successfully delivered to intended participants in some defined, but not fully controlled, context? Outcomes of interest: Degree of execution, success or failure of execution, amount, type of resources needed to implement	For adolescents (as applicable): What do you think we could have done (or done better) to help you with a good transition to adult care? What happened that prompted you to stop participating in some project activities, or continue with medications and/or medical appointments in the adult health system?
Practicality	Areas of focus: To what extent can an idea, programme, process or measure be carried out with intended participants using existing means, resources, and circumstances and without outside intervention? Outcomes of interest: Factors affecting implementation ease or difficulty, efficiency, speed or quality of implementation, positive/negative effects on target participants, ability of participants to carry out intervention activities	For adolescents (as applicable): What did the PASEO team do to help you participate in the intervention or receive the items or continue with medications and/or medical appointments in the adult health system? What would have helped you the most to continue in the intervention or to receive the elements or to continue with the medications and/or medical appointments in the adult health system but you did not receive?
Adaptation	Areas of focus: To what extent does an existing idea, programme, process or measure perform when changes are made for a new format or with a different population? Outcomes of interest: Degree to which similar outcomes are obtained in a new format, process outcomes comparison between intervention use in two populations	For adolescents: Not all the participants in the support groups shared the same characteristics (e.g. sex, gender, age, etc.). What do you think of this? Would it be better to make groups with people who are similar? During the study, as we all know, the “coronavirus” or “COVID‐19” epidemic began, which forced us to stay at home. During that period, you received calls from our team. We also offered internet chats among other things. How was the impact of coronavirus on your ability to maintain good adherence of medications? Was your schedule changed? What do you think of the new strategies we made during the epidemic? Did you like them? What do you recommend us to do or not to repeat in the future if you face a similar situation? How would we be able to improve the intervention? For healthcare personnel: How do you think the impact of coronavirus was for adolescents to maintain good adherence of the medications? Did they talk to you about the study and/or the difficulties they encountered? Based on your experience with COVID‐19, would you recommend some additional factors that we should consider for the future?
Integration	Areas of focus: To what extent can a new idea, programme, process or measure be integrated within an existing system? Outcomes of interest: Perceived fit with infrastructure, perceived sustainability	For healthcare personnel: In your opinion, how sustainable could the intervention be if it were implemented by the Ministry of Health? What concerns do you have? What would need to be changed to make it more feasible to scale? What information would you need to see before making a decision on adopt this intervention at the programmatic level?
Expansion	Areas of focus: To what extent can a previously tested programme, process, approach or system be expanded to provide a new programme or service? Outcomes of interest: Fit with organizational goals and culture, positive or negative effects on organization, disruption due to expansion component	For healthcare personnel: What would need to be changed to make PASEO more feasible to scale? What information would you need to see before making a decision on your adoption at MINSA?
Limited efficacy	Areas of focus: Does the new idea, programme, process or measure show promise of being successful with the intended population, even in a highly controlled setting? Outcomes of interest: Intended effects of programme or process on key intermediate variables, maintenance of changes from initial change	For adolescents: How has your adherence to the medications been? Why? How was your medication adherence when you were in PASEO? How is your adherence now? (What did you do or did not do strengthen adherence) Do you feel that you could continue your treatment independently after the end of the PASEO intervention? In other words, did the intervention help you? What things do you think could happen in your life that would worry you and could affect your ability to continue taking your medications on a consistent basis?

Abbreviation: MINSA, Peruvian Ministry of Health.

^a^Adapted from Ref. [37].

### 2.3 Data analysis

Data were coded by MW and BN using framework analysis [40], selected for its step‐by‐step analytical approach and usefulness in studies with specific questions among a pre‐defined sample within a limited timeframe [41]. Five transcripts were independently coded using a preliminary codebook derived from the interview guides, adding *de novo* codes as needed. Separate codebooks were created for ALWH and HIV personnel. Next, the coders and JG compared the five coded transcripts and harmonized coding by consensus after which the remaining transcripts were coded. Reports were generated for all codes, and related text across the transcripts was extracted into matrices for granular analysis. Finally, crosscutting themes were reported using illustrative quotes translated into English by JG and validated by MW. The Consolidated Criteria for Reporting Qualitative Data Checklist (COREQ, Additional File S1) was completed.

## 3 RESULTS

### 3.1 Participant characteristics

Twenty‐six ALWH participated in a focus group or in‐depth interview (*n* = 15 and *n* = 11, respectively); *n* = 4 participants were non‐responsive to invitations to participate. Fifteen of the 26 participants were male, of which 14/15 identified as cisgender; one adolescent assigned male at birth identified as transgender. Eighteen ALWH acquired HIV during early childhood and 16 had a parent die from HIV (Table 2).

**Table 2:** Characteristics of PASEO ALWH participating focus groups or in‐depth interviews (*N* = 26)

Characteristics		*N* (%)	15–18 years old (*n*)	19–22 years old (*n*)
Sex assigned at birth	Male	15 (58)	5	10
	Female	11 (42)	6	5
Sexual orientation	Homosexual	9 (35)	4	5
	Heterosexual	17 (65)	7	10
Gender	Cisgender male	14 (54)	6	5
	Cisgender female	11 (42)	5	6
	Transgender female	1 (4)	0	1
Timing of HIV acquisition	Early childhood	18 (69)	8	10
	Recent	8 (31)	3	5
Death of parent	Both parents	11 (42)	3	8
	One parent	5 (19)	4	1
Currently living with/in	Any family member	6 (23)	2	4
	Father and/or mother	8 (31)	6	2
	Group home	5 (19)	1	4
	Partner or friend	7 (27)	2	5
Lifetime history of residence in group home for children with HIV/AIDS		7 (27)	1	6

Nine key‐informant interviews were conducted with HIV programme personnel: three physicians, one nurse, two peer counsellors, two psychologists and a group home coordinator.

### 3.2 Qualitative findings

For ALWH, qualitative data are organized under two domains, Domain 1: Overall acceptability and Domain 2: Experiences with PASEO's core components. For HIV personnel, data are grouped under Domain 1: Opinions of PASEO and Domain 2: Post‐PASEO recommendations. Domain and theme(s) are presented with illustrative quotes. For adolescents, quote attributes show data source, focus group or in‐depth interview (FG/IDI), and participant/experience type (A, B, C, D, E); sex (male/female); age (years); and HIV acquisition route (early childhood [EC]/recent). For HIV professionals, due to their small number, quote attributions do not include job role to preserve anonymity.

### 3.3 Adolescents

#### 3.3.1 Domain 1, Experiences during PASEO: Overall acceptability

##### 3.3.1.1 Likes

Overall, participants were positive about PASEO, reporting a sense of camaraderie. One participant stated, “If I had not [enrolled in PASEO], I would still be without treatment; of that, I am sure” (FG‐A, female, 22, EC). Another participant summarized their experience by saying, “When I think of PASEO, the three words that come to mind are: community, teaching, help. Teaching because they taught us many things, things that I didn't know. Community because the program connected us with other people just like us” (FG‐B, female, 19, EC).

ALWH emphasized benefitting from social support; one participant said PASEO had arrived at a critical point in their life:

“…to be honest, I could say that I was totally lost—disoriented—and I didn't know how I was going to do it. Sometimes, I got so low that I felt horrible, but knowing that I had someone who supported me, that helped me, emotionally more than anything, that was what I needed most at that time.” (FG‐E, male, 19, recent)

PASEO reduced social isolation, wherein new acquaintances were non‐judgemental, and anything could be discussed without fear:

“…before [PASEO] I just stayed in my living room alone. But then I met new people and felt free to talk about things and not be judged, like any topic. They didn't judge you when you said something, whether it was right or wrong.” (IDI‐C, female, 20, EC)

##### 3.3.1.2 Dislikes

Dislikes were related to PASEO's time commitment and the changes due to COVID‐19. One participant found difficulty with the group sessions due to academic commitments, stating, “What I didn't like about PASEO was that the sessions didn't adapt very well to my schedule that I had to have for my virtual classes” (IDI‐E, male, 19, recent). Another participant disliked the virtual sessions: “The only thing I didn't like was the pandemic, because […] we couldn't meet in person, and everything was virtual. […] I liked [PASEO] before the pandemic a thousand times more” (FG‐C, female, 22, EC).

Another issue with virtual activities required by COVID‐19 was limited access to Wi‐Fi or cellular data. One participant voiced, “Well, [I didn't like] participating with video because sometimes my phone's megabytes would run out quickly, and then I couldn't use it. After my data ran out, I had to use my neighbour's Wi‐Fi when I wanted to […] get into the [online support group]” (IDI‐B, male, 20, EC).

##### 3.3.1.3 Intervention duration

Participants preferred a longer intervention period, though some thought a variable‐duration intervention responsive to the needs of each adolescent was best:

“…I didn't take the pills, or if I took them, I took them every other day; I forgot at night or in the morning because I had other things to do. I thought about other things and that's why, right? […] I don't know, I guess like [another participant] said, [the duration] depends on the person that needs the support.” (FG‐D, male, 19, EC)

Recommendations for a longer intervention duration were often framed as having more time to form/maintain social bonds with other participants; some expressed sadness that PASEO ended:

“…thinking about being close to finishing the project really affects me a lot, because I love the meetings that we have. I listen to everyone and compare myself to each person, and I realize that I'm not the only person who has suffered or had those thoughts, and I like that.” (FG‐A, female, 22, EC)

Another participant expressed, “…the end of the project is painful, and it's a little sad. But the truth is that the times that we've had have been really, really good” (FG‐A, male, 22, EC).

#### 3.3.2 Domain 2: Experiences with the six PASEO core components

##### 3.3.2.1 Component 1—Health system navigation and clinic visit accompaniment

Participants praised the support from the health promoters (“*promotora*”), with three interrelated themes emerging: immediate assistance and training; attenuating prior negative experiences with the health system; and ongoing, trust‐based working relationships. This participant discussed learning how to make clinic visit appointments independently:

“[My *promotora*] was like a guide. For example, with the [health insurance program] she told me, ‘Look, you have to be there early, you have to be persistent, and explain your situation well.’ When it was time to make appointments […] she made me do it so that I would know what to do. Then if they didn't give me the appointment or if I couldn't make the payment, she would show up and fight with who she needed to get the appointment.” (FG‐A, male, 22, EC)

Accompaniment appeared to buffer against previous bad experiences with the health system, restoring respect by health personnel: “…[in the hospital] everyone treated me really poorly the several times that I went alone. I recently went for a family planning visit, and the doctor treated me as if I was from another world. But when you have company, they respect you a little more” (FG‐B, female, 19, EC). A common view was that community health workers were “more” than project staff; they were trusted friends, “I am very grateful because [my *promotora*] was there with me even though I refused to go back to my treatment. She was there with me until I became undetectable, and I owe it all to her because she was there not only as a worker, but as my friend” (FG‐A, female, 22, EC).

##### 3.3.2.2 Component 2—Social support groups

Participants valued PASEO's support groups; however, there were disparate views on group composition. Some felt that groups comprised of all ALWH (regardless of sex, gender and HIV acquisition route) was a strength because they could learn from others with similar but different experiences: “…I learned a lot, like at least one thing I feared was not being able to have a baby, and there I learned that I can have one, because there were several guys, several girls [diagnosed with HIV] that had a baby” (IDI‐E, male, 20, recent).

Participant: “I liked how [the groups] were, I liked the group that I was in. We had great conversations, and we became friends.”

Interviewer: “So [you liked the] varied groups, with men, women, vertical transmission, or others?”

Participant: “We're all the same.” (FG‐B, female, 19, EC)

Conversely, some preferred grouping ALWH with similar experiences was beneficial, for example, those initiating ART:

“…for me, it would have been a little better if it had been the same people, those like me, to see that they were going through the same thing. Those that had just started treatment and things like that. Then, there'd be more things we have in common because, I don't know, you get to help them faster and they can help themselves too.” (FG‐E, male, 19, recent)

##### 3.3.2.3 Component 3—Screening and referral to mental health services

ALWH unanimously praised mental wellbeing support, often in the context of personal growth and acceptance of HIV:

“[The psychologist] asked me, ‘Have you really accepted yourself as you are, have you really accepted yourself?’ And that's when it struck me, I felt bad at that time, and I left the question there in the air, meditating on it for a while, realizing what the problem was. That was the problem, no? That I didn't really accept my diagnosis, I hadn't come to terms with the fact that I was an HIV carrier, that I have had to live this life.” (IDI‐A, male, 22, EC)

Another participant spoke of how PASEO helped with self‐acceptance in the face of childhood trauma: “I grew up getting bullied a lot, you know, they bullied me a lot and then made me angry at what I have, until I got here and learned many things from the people here and accepted what I have.” (FG‐B, female, 19, EC).

##### 3.3.2.4 Component 4—Resolution of acute needs

Participants spoke positively about their *promotora's* ability to address acute issues impacting ART adherence, as for this participant who lacked housing:

“…I was going to die because I was on the street, and I was going to [northern Peru]. […] [The PASEO team] took me to the hospital and that's where I also learned about the project. They saved my life because at that point I wasn't thinking about starting my medication again.” (FG‐A, male, 19, EC)

##### 3.3.2.5 Component 5—Health education and skills‐building

Participants’ experiences overlapped with thematic content for Component 3, emphasizing personal growth. A pregnant participant spoke about learning to manage her healthcare and practising self‐directed care:

“…when I was pregnant and had to go to haematology, I didn't know much about what that was for. [My *promotora*] sometimes asked the doctor questions, and the doctor told her about my haemoglobin, about what I must take, and those things. […] I usually kept quiet but ever since I saw [my *promotora*] ask the doctor questions and things like that, about her doubts, well, now I sometimes ask the doctor questions too. I learned that from her, how to speak to the doctor, because I was practically always mute in the hospital.” (FG‐C, female, 22, EC)

##### 3.3.2.6 Component 6—Individualized adherence support with DOT

For participants who received DOT, experiences were characterized as receiving helpful reminders to take ART, encouraging personal responsibility for ART adherence:

“…in my case, sometimes I didn't take my medication because I forgot to, or I lost track of time. So then with the lady's [from PASEO] calls on WhatsApp, I knew that at 8:00 PM I had to take my pill, […]. So now, like all the other girls and guys here, I think it's one's own responsibility.” (FG‐D, male, 19, EC)

For one participant, DOT—and the experience of being “confronted” by the health worker for non‐adherence—was framed as support:

“… I was very forgetful, I forgot to take my pills, I forgot my appointments, I forgot everything. […] More than anyone, the lady [from PASEO] helped me because she yelled at me every time I forgot, well she chided me. She would ask me why I forgot, and she chided me every time I forgot. I felt that she cared about me more than anyone else, more than my family.” (FG‐B, female, 19, EC)

### 3.4 HIV personnel

#### 3.4.1 Domain 1: Opinion of PASEO

##### 3.4.1.1 Overall acceptability—Improved contact with the HIV care system

HIV programme personnel similarly spoke positively about PASEO. One observation was that PASEO kept ALWH engaged in HIV care, addressing a health system gap:

“We've heard good things about the project, from participants’ family members, and we've also seen that with several of our patients, especially the difficult ones, it's been possible to keep them connected with the health system. That's really good for us because there's not a system within the hospital that does that. There's a void there, really, and [PASEO] has come to fill it.” (Participant‐7)

Another participant felt that PASEO helped ALWH independently manage healthcare: “[PASEO was] guiding them to be able to do things on their own, because they didn't even have a clue how to get to the hospitals. And now, they've restarted the treatments that they were taking before” (Participant‐3).

##### 3.4.1.2 What worked—Personalized accompaniment

Like ALWH, HIV personnel emphasized the benefit of tailoring support to the adolescents’ needs, especially when transitioning from paediatric to adult care:

“…for those who left [pediatric HIV services] to go to the other adult hospitals, [PASEO] has been really important because of the accompaniment provided. I know that each of them was given personalized support, because when you get to the adult hospital, it's different, right? There is no one there to guide you, to support you, nothing.” (Participant‐3)

The tailoring aspect of PASEO was seen to “humanize” ALWH by including non‐medical issues: “The most important thing [in PASEO] is that we are seeing the life of a human being—right? —in all its context, not only health, but also the social part, the educational part, [as well]” (Participant‐1).

##### 3.4.1.3 Support groups

Though participants spoke positively about PASEO's support groups, one potential issue noted was that ALWH could be exposed to adolescents with undesirable behaviours: “But on the negative side, [in the social support groups, ALWH] probably have access to all kinds of people and could end up falling into risky behaviours” (Participant‐4).

##### 3.4.1.4 What did not work or was lacking—Including parents/caregivers in PASEO

No aspect of the intervention elements was noted as faulty; however, one respondent felt that PASEO could be strengthened by including ALWH's parents:

“…I am very concerned about the fact, I believe, that there is no consistent work with parents […], because there are cases that I saw where parents did not really support the child or the children […], and when there were in‐person group meetings, I don't know…I feel that there should have been work with [the parents] because they are the in‐home support [for their adolescent], right?” (Participant‐6)

##### 3.4.1.5 Intervention duration

HIV personnel believed that PASEO would benefit from a longer duration, perhaps even years:

“…I think that clearly a year is pretty short, perhaps as long as it takes [is the ideal amount of time]. We could say that in this most critical time in adolescence, maybe, I don't know, 3 years, or if overdoing it, maybe 5 years. I don't have the answer, but it seems to me like one year is not enough to be able to guarantee long‐term continuity of care.” (Participant‐9)

However, the feasibility of extending the intervention if it were financed by the HIV programme was doubted by another participant. Still, even a shorter duration was felt to have benefits:

“…for the Ministry of Health, I highly doubt that they would want to do it for a year, and I really doubt that they would suddenly accept a full year. They would probably do fewer months, but really, I think that even six months would be enough, and quite important for the patient.” (Participant‐2)

##### 3.4.1.6 Specific to DOT

DOT (delivered in‐person pre‐COVID‐19 and virtually as “teledot” during COVID‐19) garnered differing opinions. Some felt that teledot was a strength and should be included in Peru's HIV Programme, regardless of PASEO: “…well, what actually caught my attention was teledot. It was a really good strategy that we also should do, apart from this project. Seeing the experience and good results that you've had, I think we should do it” (Participant‐2).

But others emphasized that DOT should be only temporary:

“HIV treatment is for life, so thinking about DOT doesn't make much sense, except in a very conjunctural situation. But really, treatment and counselling must be the aim, they must be incorporated into the lifestyle of someone living with HIV, and that's their responsibility.” (Participant‐9)

##### 3.4.1.7 Regarding COVID‐19

Participants spoke more generally about the stress that COVID‐19 had on Peru's HIV treatment programme, including hospital closures and medication stock‐outs. Still, respondents felt that PASEO buffered ALWH from the full impact of COVID‐19 on the HIV care system: “…during the pandemic, some days they didn't have access to medication [collection]. But because of the support from [PASEO], they managed to continue taking their medications” (Participant‐1).

#### 3.4.2 Domain 2—Post‐PASEO, opinions and recommendations

Regarding scaling up PASEO, two issues arose: who would implement and finance the intervention.

##### 3.4.2.1 Doubt that the public health system would implement PASEO

Concern was expressed regarding the public health system's ability to implement PASEO; however, this was expressed both as an implementation capacity and budgetary issue:

“[Implementing PASEO is] going to depend on several factors, but what is clear is that it can't be [part of the public health system's] services. It must be an external group that intervenes. In what capacity, we'll have to see, and we'll see what the budget will be as well…” (Participant‐9)

Another participant felt that implementation would need to encompass all hospitals that ALWH could transition to:

“…more than anything we'll have to see the resources, funding, to be able to have staff that do the accompaniment part, because that should be done in practically all the hospitals that care for adolescents and then transition them to adult hospitals.” (Participant‐3)

Finally, a concern arose regarding the potential for low‐quality implementation of PASEO and the negative consequences for ALWH:

“…maybe I'd worry that [the public health system] doesn't know how to [implement PASEO] properly, and there's a lot of attrition; the kids could lose confidence pretty quickly. So, if they do it and do it poorly, people are going to leave. They're going to stop following up and stop taking their pills, stop taking their treatment, you know. And that's a big risk.” (Participant‐6)

## 4 DISCUSSION

A novel CBA intervention supporting ART adherence among ALWH in Lima, Peru was highly acceptable to ALWH and HIV personnel. Among adolescents, tailored accompaniment and social support groups were especially favoured and characterized as emotionally and psychologically transformative. Notably, even participants engaging less with the intervention found PASEO valuable. Likewise, HIV personnel praised PASEO, citing favourable experiences heard from adolescents.

PASEO aimed to improve unsuppressed viremia among ALWH [35]; accordingly, the intervention focused on resolving ART adherence barriers. However, when ALWH spoke about PASEO, responses consistently centred on how each core element made them *feel* rather than the activity itself. ALWH frequently reported improved self‐acceptance as they developed emotional bonds with other ALWH. Though we could not link participants’ self‐appraisals and mental health status with HIV outcomes, an emerging area of study has begun to elucidate the negative impact of depression in adults with HIV on viral load independent of ART adherence [42]. Future research should explore the association of negative mental health states on viremia among ALWH as a potential element to improve care.

DOT for HIV has yielded mixed findings among adults [43, 44], but ALWH found DOT supportive training for independent ART adherence rather than observed verification of medication ingestion; shifting to teledot did not change this viewpoint. The favourable view of HIV teledot corroborates a study among Peruvian adults with HIV reporting perceived psychosocial outcomes as the primary benefit of DOT [45]. In that study, the stress buffer theory—positing that community support mitigates threat perceptions and improves clinical and psychosocial outcomes—may explain PASEO participants’ DOT perceptions [45, 46]. Further, our findings appear to support the positive impact found in a study of community‐based DOT in which youth with HIV experienced reduced depression and increased coping skills [47].

One reported deficit of PASEO was limited interaction with ALWH's parents/caregivers. While PASEO implicitly included ALWH's parents/caregivers, it may benefit from more explicit involvement in adolescents’ support network, and research supports involving parents to enhance ALWH's ART adherence [34, 48]. However, we note that in our sample of ALWH, over half had lost parent(s) to HIV. Therefore, while PASEO might benefit from a parent/caregiver component, our emphasis on the adolescent appears appropriate.

We wanted to understand differences in perceptions of PASEO between ALWH with early childhood and recent HIV. Practically speaking, most ALWH in Peru with recently acquired HIV also identify as a sexual and/or gender minority (SGM) [49, 50, 51] and may experience issues distinct from non‐SGM and/or ALWH living with HIV from birth [52]. While one participant suggested that separate groups for those with early childhood versus recently acquired HIV could help to share common experiences, overall, ALWH liked hearing experiences different than their own, endorsing the mixed‐group approach. Importantly, no SGM reported feeling marginalized. This finding might be explained by the balance between individual CBA tailored to each ALWH's specific needs and the support groups which focused on themes that most ALWH could relate to regardless of their background. From an implementation perspective, forming groups with open membership may be easier than multiple, membership‐specific; further research should explore the pros and cons of both approaches.

If PASEO is found to be efficacious in optimizing ALWH's ART adherence, its impact will be constrained unless it is adequately scaled. HIV personnel voiced concerns regarding financing the intervention and had discrepant views on its duration. These findings speak to future research steps for PASEO, especially an economic analysis to understand PASEO's economic viability.

As a small, pilot, qualitative study, our findings are limited to the experiences of the study participants and cannot be generalized. We attempted to recruit a diverse group of adolescents, but only one transgender female participated; future studies must increase SGM participation. Further, we were unable to discuss ALWH's perceptions on viral load changes during PASEO because COVID‐19 disrupted viral load testing in Peru, and only baseline results were available [35]. Finally, the near uniformly positive responses from ALWH could be due to social desirability bias; however, this was minimized by collecting data after the intervention ended at the final study contact.

## 5 CONCLUSIONS

A multicomponent CBA intervention addressing physical, mental, reproductive and psychosocial wellbeing to support ALWH's ART adherence in Peru was highly acceptable. Future research should determine the efficacy and economic impact of the intervention.

## COMPETING INTERESTS

The authors declare that they have no competing interests.

## AUTHORS’ CONTRIBUTIONS

MFF conceived the study. SS, LL, MW, KR, AR, LS, JTG and HS designed the intervention, which was coordinated by MW and implemented by AR, LS, RAE, AL and HS. LK, EM, ES and CB provided feedback on intervention design and recruitment. JTG led the qualitative analysis. MW collected qualitative data, which was analysed by JTG, MW and BN. JTG, MW, BN and MFF drafted the manuscript, which was reviewed, edited and approved by all authors.

## FUNDING

This research was entirely supported by the National Institute of Allergy and Infectious Diseases of the National Institutes of Health under award number R21 AI143365.

## Data Availability

The data that support the findings of this study are available on request from the corresponding author. The data are not publicly available due to privacy or ethical restrictions.
